# Potential Clinical Significance of Overall Targeting Accuracy and Motion Management in the Treatment of Tumors That Move With Respiration: Lessons Learnt From a Quarter Century of Stereotactic Body Radiotherapy From Dose Response Models

**DOI:** 10.3389/fonc.2020.591430

**Published:** 2021-02-09

**Authors:** Anand Mahadevan, Bahman Emami, Jimm Grimm, Lawrence R. Kleinberg, Kristin J. Redmond, James S. Welsh, Robert Rostock, Eric Kemmerer, Kenneth M. Forster, Jason Stanford, Sunjay Shah, Sucha O. Asbell, Tamara A. LaCouture, Carla Scofield, Ian Butterwick, Jinyu Xue, Alexander Muacevic, John R. Adler

**Affiliations:** ^1^ Department of Radiation Oncology, Geisinger Cancer Institute, Danville, PA, United States; ^2^ Department of Radiation Oncology, Loyola University Medical Center, Chicago, IL, United States; ^3^ Department of Radiation Oncology and Molecular Radiation Sciences, Johns Hopkins University School of Medicine, Baltimore, MD, United States; ^4^ Department of Radiation Oncology, Helen F. Graham Cancer Center, Christiana Care Health System, Newark, DE, United States; ^5^ Department of Radiation Oncology, Thomas Jefferson University, Philadelphia, PA, United States; ^6^ Department of Radiation Oncology, New York University, New York City, NY, United States; ^7^ European Cyberknife Center Munich, Munich, Germany; ^8^ Department of Neurosurgery, Stanford University School of Medicine, Stanford, CA, United States

**Keywords:** normal tissue complication probability, dose response, tracking, stereotactic body radiation therapy, radiosurgery

## Abstract

**Objective:**

To determine the long-term normal tissue complication probability with stereotactic body radiation therapy (SBRT) treatments for targets that move with respiration and its relation with the type of respiratory motion management (tracking *vs*. compression or gating).

**Methods:**

A PubMed search was performed for identifying literature regarding dose, volume, fractionation, and toxicity (grade 3 or higher) for SBRT treatments for tumors which move with respiration. From the identified papers logistic or probit dose-response models were fitted to the data using the maximum-likelihood technique and confidence intervals were based on the profile-likelihood method in the dose-volume histogram (DVH) Evaluator.

**Results:**

Pooled logistic and probit models for grade 3 or higher toxicity for aorta, chest wall, duodenum, and small bowel suggest a significant difference when live motion tracking was used for targeting tumors with move with respiration which was on the average 10 times lower, in the high dose range.

**Conclusion:**

Live respiratory motion management appears to have a better toxicity outcome when treating targets which move with respiration with very steep peripheral dose gradients. This analysis is however limited by sparsity of rigorous data due to poor reporting in the literature.

## Introduction

In the past 25 years (1–9) with the experience gained from millions of patients, radiosurgery has evolved into an ever more effective treatment of tumors throughout the body (10–15). Despite the broad impact of radiosurgery, the underlying technical principles have been simple: 1) highly accurate overall targeting including motion management, 2) steep-dose gradients, and 3) image guidance or strict immobilization. Nevertheless, due to its inherent nature of high fractional radiation doses and the often-close proximity of critical anatomy adjacent to lesions undergoing radiosurgical ablation, even small inaccuracies risk complications. Although there exists a wide variety of targeting methods and corresponding accuracy of radiation devices being used to administer ablative radiosurgery, it has not been demonstrated to date that such differences are clinically significant. Such an analysis is made especially difficult by the fact that published datasets tend to be sparse with rather limited follow-up, while some complications can occur late, making it particularly challenging for us to estimate long term risks. Phase III clinical studies would be the ideal solution but usually such trials are designed to evaluate new cancer drugs or treatment modalities instead of toxicity dose response models. Now, after 25 years of clinical experience, and with the recent arrival of automated clinical tools for quantifying dose-response outcomes (16, 17) in terms of normal tissue complication probability (NTCP) models, it might be possible to finally quantify the late-effect complications from radiosurgery, and thereby better define the risk/benefit ratio of this important therapy.

More than five decades ago, radiosurgery itself, through Gamma Knife (Elekta Inc., Stockholm, Sweden), radiosurgery was conceptualized by Dr. Lars Leksell (1, 2). Meanwhile, 30 years ago, the Winston-Lutz technique for measuring stereotactic accuracy was described (3), making it then much simpler to precisely measure the accuracy of stereotactic linear accelerators (4–7). Twenty-five years ago, the first extracranial radiosurgery cases were published (8), and the first robotic stereotactic treatment was performed using real time image-to-image correlation (CyberKnife Accuray Inc., Sunnyvale, Ca, USA) (9). Shortly thereafter continuous live tracking of respiratory motion (Synchrony - Accuray Inc., Sunnyvale, CA, USA) was introduced into clinical practice. Over time many more radiation devices have been utilized for radiosurgery, ranging from protons, to heavy ions, and a number of modified linear accelerators (e.g., TrueBeam, Synergy, Novalis, Edge, ViewRay) have incorporated stereotactic-like delivery capabilities using varying methods, most notably pre-treatment cone beam CT or MRI. Today there are many technologies used to localize targets in the stereotactic space in therapeutic radiation devices. These technical differences are most striking when it comes to not only visualizing the target but also for compensating for motion, especially with respiration. While it is not yet practical to compare outcomes between different treatment devices, a comparative analysis is needed to better understand and quantify the GENERAL importance of overall targeting accuracy when treating targets that move during respiration. Fortunately, automated software tools now make it practical to continuously capture treatment parameters and outcomes data as part of the normal clinical workflow. In doing so, it is now possible to better understand the relative merits of the different stereotactic platforms. Given these new analytical tools, the goal of the present study is to evaluate, and quantify, the dosimetric influences due to target motion and its compensation, on NTCP of targeting errors associated with current generation of radiosurgical devices using a modeling-based approach.

## Methods and Materials

A PubMed search was performed to identify all published data that could be used to construct NTCP models for targets that move with respiration, to compare techniques with continuous motion tracking to other stereotactic systems without this capability using alternate methods to account for motion (compression, gating etc.), in the range of doses in which complications occurred. A model-based approach was used to view the data, and the final physical dose comparisons were made with all comparative thresholds less than 10 Gy per fraction. To achieve relevant and unbiased comparisons, outcomes were considered in the dose range in which complications were reported, with a threshold adjusted to the level that enabled unambiguous comparisons in terms of physical dose. Dose volume histogram (DVH) levels Dx were analyzed, where Dx denoted the dose in the DVH corresponding to volume x. Prior to dose-response modeling for each critical structure, all Dx values were converted to a common fractionation using the linear quadratic (LQ) model (18). Logistic (19) or probit (20) dose-response models were fitted to the data using the maximum-likelihood technique (21) and confidence intervals were based on the profile-likelihood method (22) in the DVH Evaluator (DiversiLabs, LLC, Huntingdon Valley, PA) (16, 17). The endpoint of grade 3 or higher complications, according to the Common Terminology Criteria for Adverse Events (CTCAE) (23), version 3 for chestwall and duodenum, and version 4 for aorta/major vessels and small bowel was chosen for clinical relevance. Tests of significance were performed with 2x2 contingency tables and Fisher’s Exact Test (24) and Cochran-Mantel-Haenszel Test (25). Detailed methods and materials, as well as discussion of caveats for each study are presented in the source references (26–29). A typical linear accelerator off-axis profile curve also was considered to assess the potential effect of overall targeting accuracy on the dose distribution.

## Results

**Figure 1:**
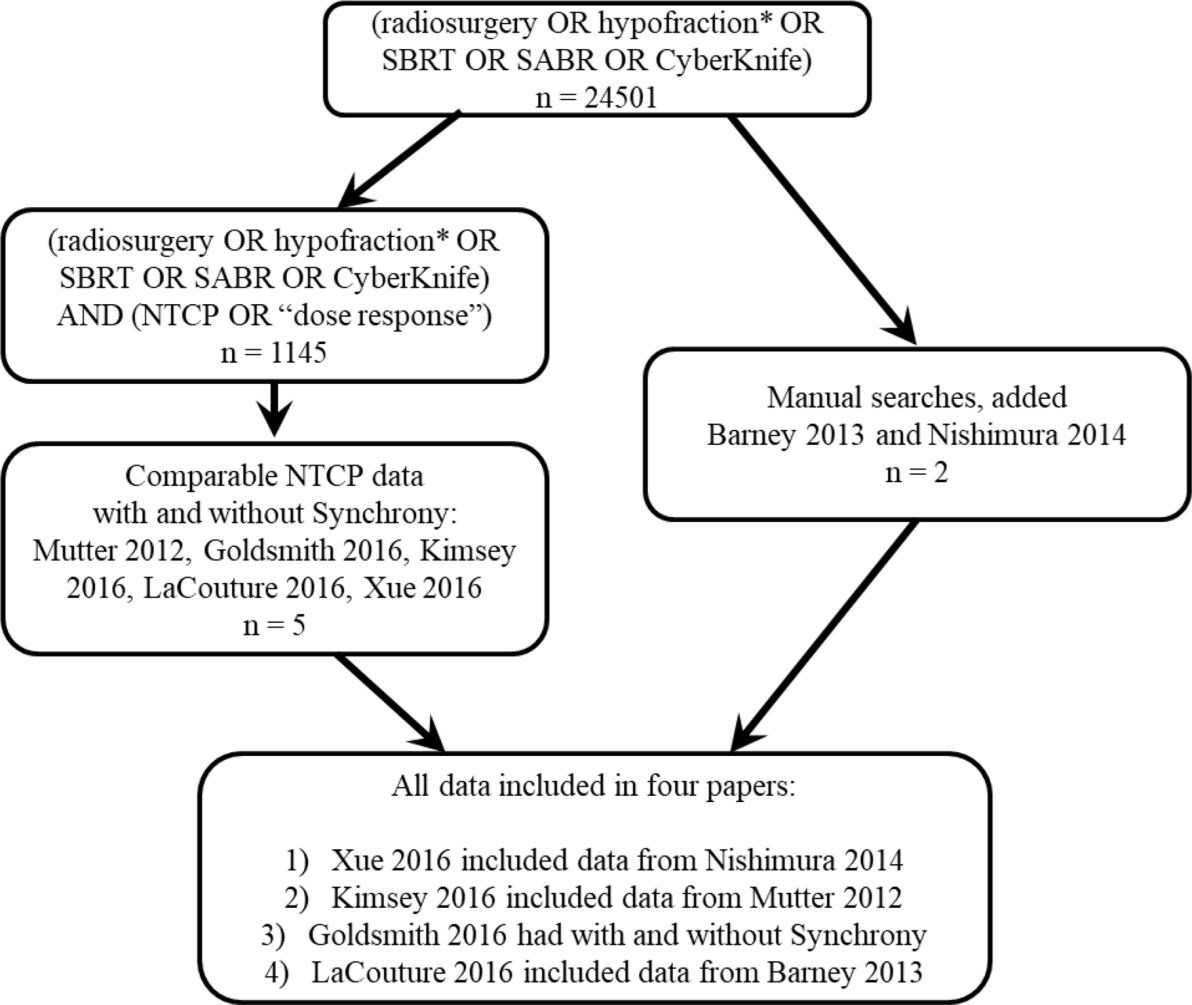
PRISMA diagram showing that less than half a percent of the published stereotactic body radiation therapy (SBRT) literature was found to have normal tissue complication probability (NTCP) models, and only four papers were found (26–29) that had enough detail to compare NTCP dose-response with and without motion tracking.

**Figure 2:**
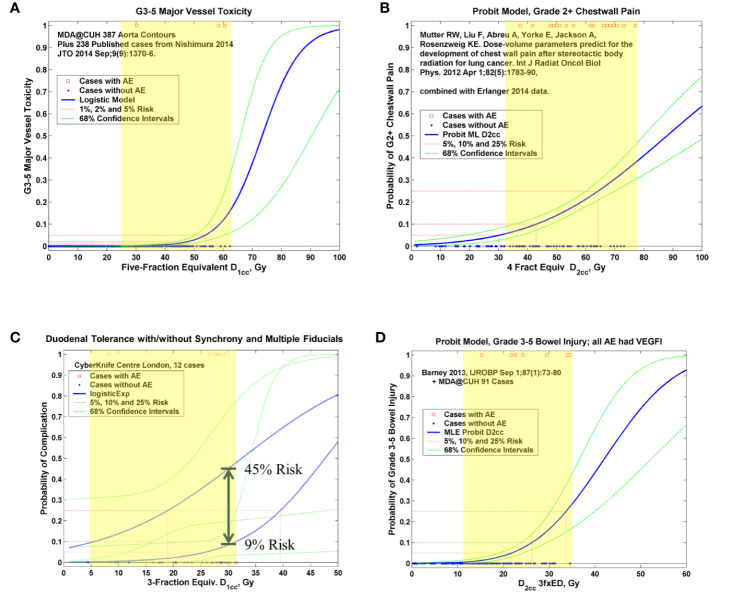
Pooled logistic (19) or probit (20) models for aorta and major vessels **(A)**, chest wall **(B)**, duodenum **(C)**, and small bowel **(D)**. In each graph the red squares represent planned critical structure doses at which complications occurred and the blue dots represent planned doses that did not result in a complication, on a per-patient basis. The solid blue curve is the maximum-likelihood fitted logistic or probit model (26–29). The dashed green curves are the confidence intervals based on the profile-likelihood method (22). The yellow highlighting shows the region of comparison, as summarized in **Table 1**. AE, adverse event; NfxED, N-fraction equivalent dose; DVH, dose volume histogram; Dx, DVH level corresponding to volume x; VEGFI, vascular endothelial growth factor inhibitor; MLE, maximum-likelihood estimate.

**Figure 3:**
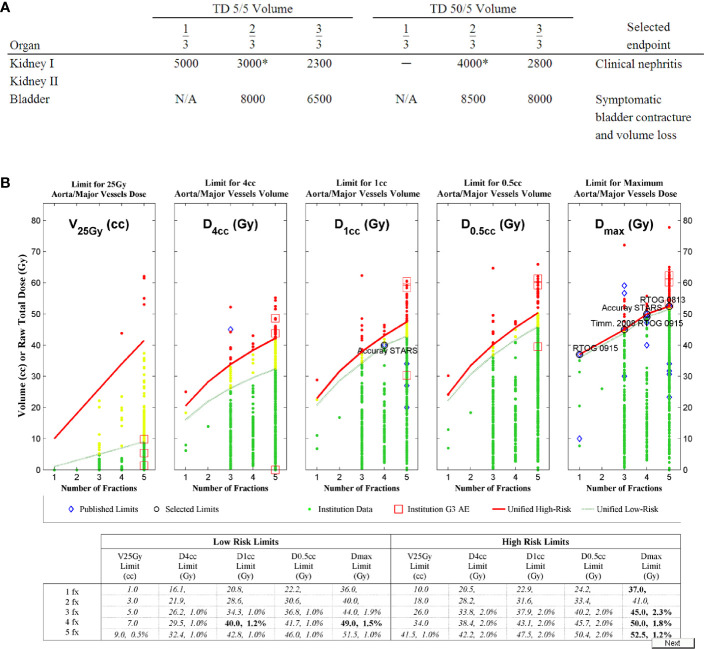
The Emami table **(A)** (34, 35) for conventional fractionation as compared to **(B)** the dose-volume histogram (DVH) Risk Map for aorta dose tolerance in 1 to 5 fractions, with estimated risk levels from the model in **Figure 2A** (26) as the second number in each cell of the table, when available. Like the Emami table, the DVH Risk Map has both low-risk limits and high-risk limits. The DVH Risk Map additionally plots the data graphically and has a separate row for each degree of fractionation. Dashed green lines represent the low-risk limits and green dots represent individual patient data that is below the low-risk limits. Red lines represent high-risk limits and red dots represent individual patient data that is above the high-risk limits. Yellow dots are patient data between low- and high-risk limits. Red squares represent doses at which grade 3 (G3) or higher adverse events (AE) occurred. Blue diamonds represent published dose tolerance limits (36–37), and representative well-established limits have been circled and labeled.

Over 20,000 papers from a PubMed search (radiosurgery OR hypofraction* OR SBRT OR SABR OR CyberKnife) until January 2019 were screened, as shown in **Figure 1** in terms of a preferred reporting items for systematic reviews and meta-analyses (PRISMA) diagram. Only four papers were found that provided dose, fractionation, and volume information per patient with toxicity outcomes for targets that move with respiration, for either continuous motion tracking or other radiation therapy techniques, in sufficient detail that dose-response model comparisons could be generated (26–29). This along with data pooled from the original sources that used the internal target volume (ITV) approach (30–32), are summarized in **Figure 2**. To alleviate LQ conversion effects, the models were created in the fractionation nearest the bulk of the high-dose data: five fractions for the aorta/major vessel model due to the tendency to fractionate more when close to such an important structure (26, 30), four fractions for the chestwall model since most of the data was in 3–5 fractions (27, 31), three fractions for the duodenal model since almost all of the data was in three fractions (28), and three fractions for the small bowel model (29, 32). The range of doses for comparison, corresponding to the range at which complications occurred, is highlighted in yellow. It may be seen that in each graph of **Figure 2** the data extends beyond 10 Gy per fraction, but the lower edge of the yellow band is less than 10 Gy per fraction in each graph to ensure comparative thresholds are all in a range where the LQ model is most acceptable (33). With the comparative thresholds set as shown in **Figure 2**, continuous live motion management had an average of 10 times lower risk of complications than alternative techniques, which do not fully account for respiratory motion (**Table 1**). Part of the classic Emami table (34, 35) is shown in **Figure 3A** and the automated output of the DVH Evaluator for the pooled aorta/major vessel data (26, 30) is compared in **Figure 3B** with dose constraints from numerous sources (26, 36–38) overlaid; see **Appendix Figures A1, A2** for a general description.

**Table 1:** Comparison of outcomes in the shaded dose range of complications from **Figure 2**.

	Total patients in dose range		Grade 3 or higher complications		Fisher exact
	CyberKnife with synchrony	Linac or no synchrony	CyberKnife with synchrony	Linac or no synchrony	p-value
Aorta/major vessels D1cc	111	133	0	3	0.253
Chest wall D2cc	25	114	0	19	0.024
Duodenum D1cc	32	11	2	3	0.097
Small bowel D2cc	47	65	0	7	0.021
Total	215	323	2	32	
**Average risk**			**1%**	**10%**	p<0.0002*

*The cumulative data p-value was 0.000006 via Fisher Exact Test (24) and was 0.00017 when calculated with Cochran-Mantel-Haenszel Test (25); both calculations of this p-value are less than 0.0002.

The chest wall graph (**Figure 2B**) shows the risk of grade 2 or higher complications, because in this situation the DVH atlas only provided dose-volume data for 27 grade 2 or higher complications (31). However, the accompanying text of the Mutter et al. (31) manuscript clarified that 19 of them were grade 3 or higher, so only these 19 grade 3 cases were scored as complications for the actual comparison in **Table 1**.

## Discussion

### Overall Targeting Accuracy Including Motion Management

Meaningful overall targeting accuracy for lung and abdominal tumors should include not only system mechanical alignment, but also motion management of moving targets (41–44). The American Association of Physicists in Medicine (AAPM) task group (TG) 135 report (41) uses such a comprehensive definition as to even mention effects of fiducial geometry as part of overall accuracy. For tracking modes applicable to moving targets, the end-to-end test for robotic radiosurgery is measured with a moving phantom, thus overall targeting accuracy includes aspects of motion management (41). From the clinical reports (26–32) it was not possible for us to analyze each aspect of overall targeting accuracy separately, but from the data in **Table 1** it appears that live motion management may be one of the largest factors for toxicity outcomes during respiratory related SBRT treatments. Limitations of available data including short follow-up and low number of toxicity events preclude detailed analysis currently, and this warrants further study and characterization with more and higher quality data in future studies.

### Dose Gradient and Overall Targeting Accuracy

**Figure 4:**
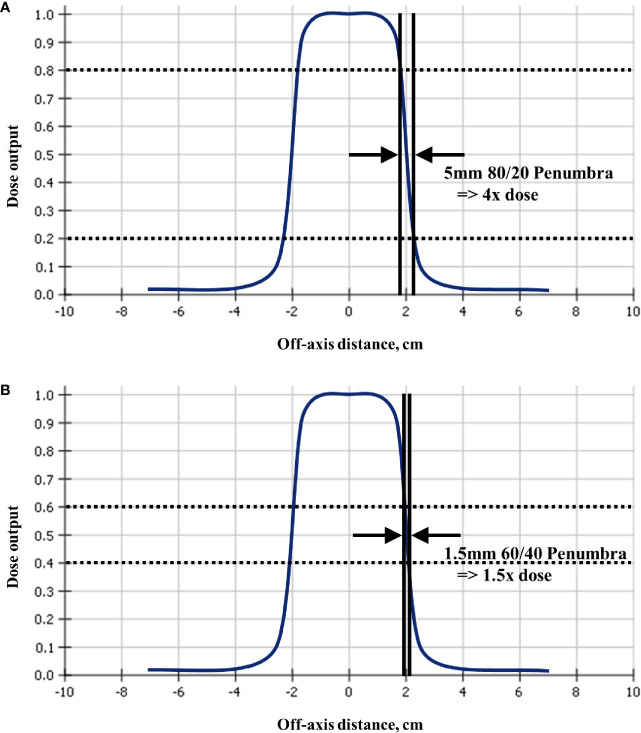
Since radiosurgery uses beam collimation to block adjacent critical structures from receiving high dose, a typical linear accelerator off-axis profile for a 4cm collimator shows that a targeting error of **(A)** 5mm could result in four times and **(B)** 1.5mm could result in 1.5 times, higher than planned dose to the critical structure. This figure is a simplistic single-beam illustration to explain the dose gradient concept, whereas more realistic examples with multiple beams are shown in **Appendix Figure A4**, where 2mm away from the surface of the spinal cord the dose is 50% higher, and in **Appendix Figure A5** where 1.5mm away from the surface of the chiasm the dose is 50% higher.

Conventional three-dimensional and intensity modulated radiation therapy can allow large volumes of critical structures to receive a substantial dose (34, 35) because of the extended fractionation. In contrast, the ablatively high dose per fraction used in SBRT requires much smaller volumes of critical structures to be tightly constrained (37, 45) which is usually achieved by highly optimized plans with steep dose gradients. When critical structures are close to the target, this large change in dose over very small distances increases the importance of overall targeting accuracy. **Figure 4** which depicts the beam profile of a typical linear accelerator 4 cm diameter field, to illustrate the concept, and **Appendix Figures A4, A5** have patient plans exhibiting this stark reality. As seen in **Figure 4**, the distance from 80 to 20% of full dose is a mere 5mm while the distance from 60 to 40% is only 1.5mm. Therefore a 5mm targeting inaccuracy in a pancreas SBRT plan could result in 4 times higher Dmax to the duodenum from some beams (**Figure 4A**), while a 1.5mm targeting inaccuracy in a spine plan could result in 1.5 times higher Dmax to the spinal cord from some beams (**Figure 4B**). This is particularly relevant in cases involving targets that inevitably move with respiration, as in the example in **Appendix Figure A3**. The simplistic single-beam example in **Figure 4** may be helpful to illustrate the dose gradient concept, but actual clinical SBRT plans often have many beams or multiple arcs with a much more complex dose distribution. However, in highly optimized plans, to achieve the steepest possible dose gradient between targets and critical structures, the planning system algorithm inherently may drive beam and segment selection towards tangential arrangements in the vicinity of these interfaces, such that the calculated dose may approach this ideal. Two clinical examples are the 70 beam non-isocentric plan in **Appendix Figure A4** where planned dose near the cord changes by as much as 50% in 2mm, and the 40 beam non-isocentric plan in **Appendix Figure A5** where planned dose near the chiasm changes by as much as 50% in 1.53mm. This can be a factor in few or many beams and planning system optimizers ideally aim to achieve a steep dose gradient.

Toxicity outcomes depend not only on prescription, but also on the dose distribution received by the critical structure. Unfortunately this level of detail is rarely reported per patient in the literature, as evidenced by the PRISMA diagram in **Figure 1**, therefore we were only able to perform a grouped comparison of a few systems. There is a great need in the published data for dose-response models from every type of delivery system, including compression, tracking, breath-hold, gating, MRI guidance, etc. The literature review only revealed data for a single system that used tracking, where tracking is defined as a system that does not need gating because the delivery system continuously moves with the tumor while the beam is on, based on a breathing model from internal imaging of the tumor or a surrogate (e.g., fiducials).

### Additional Factors Potentially Affecting Outcomes

Volume effects are another potential explanation for the large differences among systems as observed in **Figure 2** and **Table 1**. The non-tracking patients are usually treated with an ITV that includes an additional volume expansion to account for anticipated tumor motion. This technique usually results in a larger planning target volume (PTV) than would be used for a tracking system, with consequently higher dose/volume to the adjacent organs at risk. The dose-response methodology partially accounts for this, in that the yellow shaded dose ranges of **Figure 2** have planned doses from both types of systems for fair comparisons. However, due to sparsity of data, a simple binary threshold was used to generate **Table 1**, whereas future studies with more data could better explore finer dose/volume thresholds with and without ITV and for all the delivery systems.

The example of duodenal toxicity analysis where all patients were treated in the same institution with the same delivery system but with different tracking techniques is an interesting example deserving a separate discussion, but for many reasons still does not completely separate the underlying causes. Even when mechanical accuracy of the system was the same for all patients, the cohort with multiple fiducials enhancing live motion tracking had lower risk of toxicity in the high dose region (**Figure 2C**). Some additional factors that may have contributed to the results in this dataset are: patient selection, staging, and prognosis of the patients, age and other patient characteristics, comorbidities, tumor size, proximity of tumor to the duodenum, quality of the motion surrogate, and so forth. Compliance with dose/volume constraints varies from one case to another, and this is largely accounted for in the dose-response models, but more data is needed to fully understand the dose-volume relationships in improved models. In summary, many other factors potentially affecting outcomes may have played a role, and a large amount of high-quality data is needed to perform the multivariate analysis that would be required to determine which are most important.

### Dose Escalation–Conventional Fractionation to Stereotactic Body Radiation Therapy

In one extreme if we deliver zero dose, then all treatment modalities and machines would be the same. As we escalate dose to the highest feasible levels to achieve better tumor control, the differences among techniques become relevant. Radiosurgery deliberately escalates this concept to the extreme, where daily doses an order of magnitude higher than a conventional 2 Gy/day are becoming routine. Therefore, the most relevant comparisons should be made in the high dose range using dose response models, because even when the prescription is the same for all patients, the critical structures may receive a wide range of dose distributions. Furthermore, animal models have shown that when the dose is sufficiently high to the organs at risk, it results in complications regardless of technique (49, 50); zero dose is not of clinical interest and neither is this excessive dose on the other extreme. The goal is to find the ideal dose with highest likelihood of tumor control subject to normal tissue tolerance; future studies should include sufficient data to refine this range. This is particularly true for moving targets.

### Respiratory Motion Management

Unlike intracranial radiosurgery, extracranial targets can move. Unpredictable (bowel gas, patient twitching) and predictable respiratory motion are “the Achille’s Heel” of SBRT targeting. Any spatial inaccuracies may cause the sharp beam profiles to bring normal tissue to dangerously high dose levels and are associated with a much greater risk of long-term toxicity. In this study we have observed from dose-response modeling that continuous live motion management (e.g., Synchrony) will theoretically maintain both a better dose and sharper fall off when treating moving targets with respect to adjacent organs at risk.

### Background and Perspective

To remain impartial in research it is necessary to be open-minded to views differing from the original hypothesis. Historically, it is important to note that eminent radiobiologists like Jack Fowler began their life’s work studying ***hyper***fractionation (51–53), which is the complete opposite of SBRT’s ***hypo***fractionation. Within 6 years of studying ***hyper***fractionation for prostate, Fowler began to investigate the possibility of ***hypo***fractionation (54, 55), and not until studying pooled models of many clinical outcome studies did he begin to strongly propose ***hypo***fractionation (56).

The discovery of the radiobiologic importance of vascular damage also began from research in the opposite direction of hypofractionation. Conventional wisdom was that a high initial dose like 10 Gy per fraction could increase blood flow through reoxygenation and overcome hypoxia, to be then followed by a more effective conventional course of oxygenated radiotherapy. Early investigations from Song *et al*. began with goals like, “reoxygenation of hypoxic tumor cells during the course of treatment is considered one of the major factors responsible for the success of clinical radiotherapy” (57), but instead found that tumor cells continued to die days after the single high dose of radiation. This was later attributed to “substantial damage to the tumor vasculature” (58) but during the 1970s it was not feasible to deliver such a high dose per fraction safely to humans. More than 30 years later, a paper discussing hypoxia and SBRT (59) prompted several letters to the Editor (60–62). Suddenly fitting the pieces together, Dr. Song fully realized that instead of reoxygenating the hypoxic tumor cells, the high single dose would predominantly “cause considerable vascular damage throughout the tumors and deplete oxygen supply to tumor cells, leading to deoxygenation of tumor cells rather than reoxygenation of hypoxic tumor cells” (62). A full summary of the vascular damage secondary cell death hypothesis was immediately forthcoming (63), which has led to lively discussions in the literature (64–73) and has now become a HyTEC paper (74).

A third example of discovery from the opposite direction of the original goal is the present study, which began as a quest to achieve submillimeter end-to-end accuracy on any stereotactic linac (75) but differences in outcomes among the various systems were observed. Soon, the tests validated that continuous live respiratory motion management indeed can achieve submillimeter end-to-end targeting accuracy and excellent dosimetric accuracy in a wide variety of situations (44, 76, 77), and the resulting clinical outcomes comparatively appear exceptionally good, as summarized in this study. This was also seen in the series of publications in the NTCP for SBRT issue of Seminars in Radiation Oncology (78). This highlights the importance that the dose distributions and outcomes for all patients should be prospectively captured and analyzed so we can continually assess the relative merits of every treatment modality and device in the most unbiased and objective manner possible, always open to the possibility that we may find the opposite of our initial objective.

The current status of SBRT is still lacking adequate data, with many potential factors affecting outcomes that are not yet fully explained, therefore it is particularly important for all to remain unbiased.

### Reducing Bias in Analyzing Comparative Efficacy

To avoid potential bias regarding the models, the final comparisons in **Table 1** were done in terms of physical dose. The comparison threshold was first based on the dose range in which complications occurred in **Figure 2**, and then alternate fractionations were considered. In the chest wall dataset in **Figure 2B** (31) the lowest D2cc that corresponded to a complication was 33 Gy in three fractions, which corresponds to 37.4 Gy in four fractions if α/β=3 Gy. The threshold for comparison was set at 1.1 Gy/fraction less than this, or 33 Gy in four fractions, which is a lower biologically effective dose than 33 Gy in three fractions for any conceivable BED model. Using the slightly lower threshold has the additional advantage of not biasing the results by using only the range of data with complications; it widens the range of comparison to reduce bias. The lowest Dx corresponding to a complication for each of the other critical structures (**Figures 2A, C, D**) was all in terms of physical dose in the fractionation used in the model, so no conversions were needed. A reduction of 1 Gy/fraction less than the Dx of the complication was used for the threshold, to remain consistent with the chest wall comparisons, and to widen the range of comparison to increase reduce bias and generalizable to routine clinical practice. With the dose range of comparison as shown in **Figure 2**, techniques with live respiratory motion management and multiple fiducials had 10 times lower risk of grade 3 or higher complications than linac base radiosurgery without respiratory tracking, for targets that move with respiration.

### Sparsity and Quality of Data

**Figure 5:**
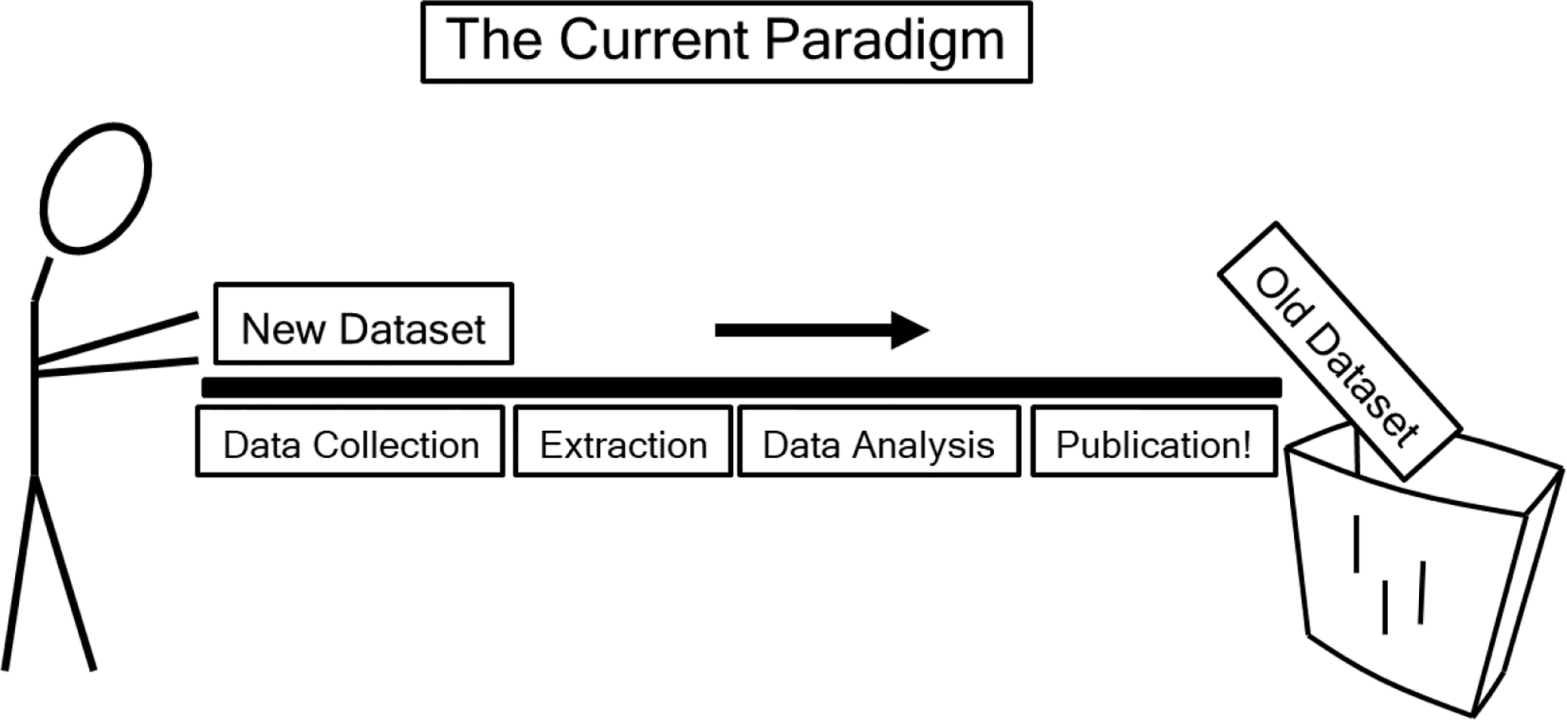
“The current (data-loss) paradigm” as depicted in QUANTEC (46), reproduced with permission. “Data are effectively lost to the wider scientific community after publication. Capturing key datasets in query-able data repositories would accelerate the discovery of causative factors and increase the accuracy of parameter estimates” (46).

More than a million patients have been treated with Gamma Knife (14) radiosurgery; similarly, more than a million patients have been treated with CyberKnife (15) technology, and countless others have been treated on stereotactic linear accelerators. Sparsity of adequately reported data is not due to lack of patients, it is because our field has not adopted the data pooling culture as recommended by QUANTEC (46), and because we have not adopted the improved reporting standards as recommended by QUANTEC (47) (**Figure 5**). Most papers report the prescription dose without providing the dose distribution to the critical structures and associated toxicity outcome, so there is no possible way to generate NTCP from most papers published in the current literature. To capture this data automatically as part of the normal clinical workflow, a device already exists that has food and drug administration (FDA) 510(k) clearance, the DVH Evaluator (16, 17), with output such as **Figures 2** and **3B** generated automatically. If such automated tools were routinely used then the data from every patient could provide large amounts of extremely useful information so that differences of the various devices with respect to image guidance and respiratory motion management and its effect on clinical outcomes could be studied published.

### Limitations

Systematic, standardized, and uniform data collection of treatment parameters and complications are lacking. Technically, physics measurements have shown that the even with respiratory motion management and in lung material, ray tracing algorithm can be off by up to 120%, although improved measurements by some of the same authors later matched the Monte Carlo algorithm within 3% (77, 79). Similarly, when motion management techniques were used without multiple fiducials and synchrony tracking, the risk of duodenal complications was found to be five times higher (28). These and several other factors have the potential to affect outcomes, including fiducial geometry, imaging technique and frequency, comorbidities, patient selection, and multidisciplinary combined modality therapies including systemic and immune therapies. Consistently defined endpoints need to be used in prospective studies in many institutions accounting for the time to occurrence of late effects to systematically create fair and unbiased comparisons of all treatment modalities and devices. It is apparent that the 68% confidence intervals were used in **Figure 2** instead of 95%, which is indicative of wider spread of uncertainty due to the sparsity of the data, and even the 68% confidence intervals have a large spread. Future studies with more patients are needed with 95% confidence intervals to reach a better understanding of the uncertainty of the analysis, for all delivery systems and motion management techniques, particularly seeking to quantify the relative importance of the many factors affecting outcomes. Technology continues to improve from many vendors and results from the latest capability should be analyzed to ensure fair comparisons of the best achievable outcomes. Our encouraging preliminary results presented from the practice of SBRT for 25 years has taught us that we need to conduct more extensive studies accounting for many of these confounding variables and practice rigorous reporting and data collection as lessons for the immediate future.

## Conclusions

Accurate overall targeting including rigorous live respiratory motion management, is crucial for safe high dose per fraction stereotactic ablative radiotherapy, to the extent that potential risk among different radiosurgical modalities can vary by a factor of 10. From the data in **Table 1** it appears that live motion management may be a large factor for toxicity outcomes during respiratory related SBRT treatments. Our encouraging preliminary results presented here from the practice of SBRT for over 25 years has taught us that we need to conduct more extensive studies accounting for many confounding variables and practice rigorous reporting and data collection as lessons for the immediate future. A data pooling culture and improved reporting standards, as recommended by QUANTEC, are desperately needed in the field of radiation oncology particularly pertaining to stereotactic body radiotherapy. Outcomes from all SBRT treatment devices in many institutions should be continually updated and published to better understand and quantify risk benefits and refine prediction models.

## Data Availability Statement

The original contributions presented in the study are included in the article/**Supplementary Material**. Further inquiries can be directed to the corresponding author.

## Author Contributions

All authors contributed to the article and approved the submitted version. BE inspired the paper at his Luther Brady lecture at ACRO 2016. JG, SA, TL, and JX collected, analyzed, and published the source datasets and began the manuscript. LK, KR, JW, EK, KF, JS, SS, CS, and IB contributed to the revisions ensuring technical accuracy. AMa, BE, RR, AMu, and JA are the senior authors of the work and made major edits especially toward completion of the work.

## Conflict of Interest

JG reports grants from Accuray and NovoCure, outside the submitted work. JG also has a patent DVH Evaluator issued. JA reports conflicts from Accuray, Varian, Zap Surgical, outside the submitted work. AM reports conflicts from Accuray and Varian outside of this submitted work.

The remaining authors declare that the research was conducted in the absence of any commercial or financial relationships that could be construed as a potential conflict of interest.
