# Supplementary material for: Potential Clinical Significance of Overall Targeting Accuracy and Motion Management in the Treatment of Tumors That Move With Respiration: Lessons Learnt From a Quarter Century of Stereotactic Body Radiotherapy From Dose Response Models
Source: Front Oncol. 2021 Feb 9;10:591430. doi: 10.3389/fonc.2020.591430 (PMC7900559; doi:10.3389/fonc.2020.591430)
Supplement: Supplementary file 1 [file DataSheet_1.docx]

**Appendix**

Risk estimates of the Emami limits (34) were from a combination of expert opinion and the eye-fitted Burman models (35) as depicted in Fig. A1. With improved amount of data, the DVH Risk Maps can now be exclusively created from maximum-likelihood parameter fitted (21) clinical outcomes data, as in Fig. A2, although more data is still needed to strengthen the accuracy of the risk estimates, especially from each different treatment device and technique.


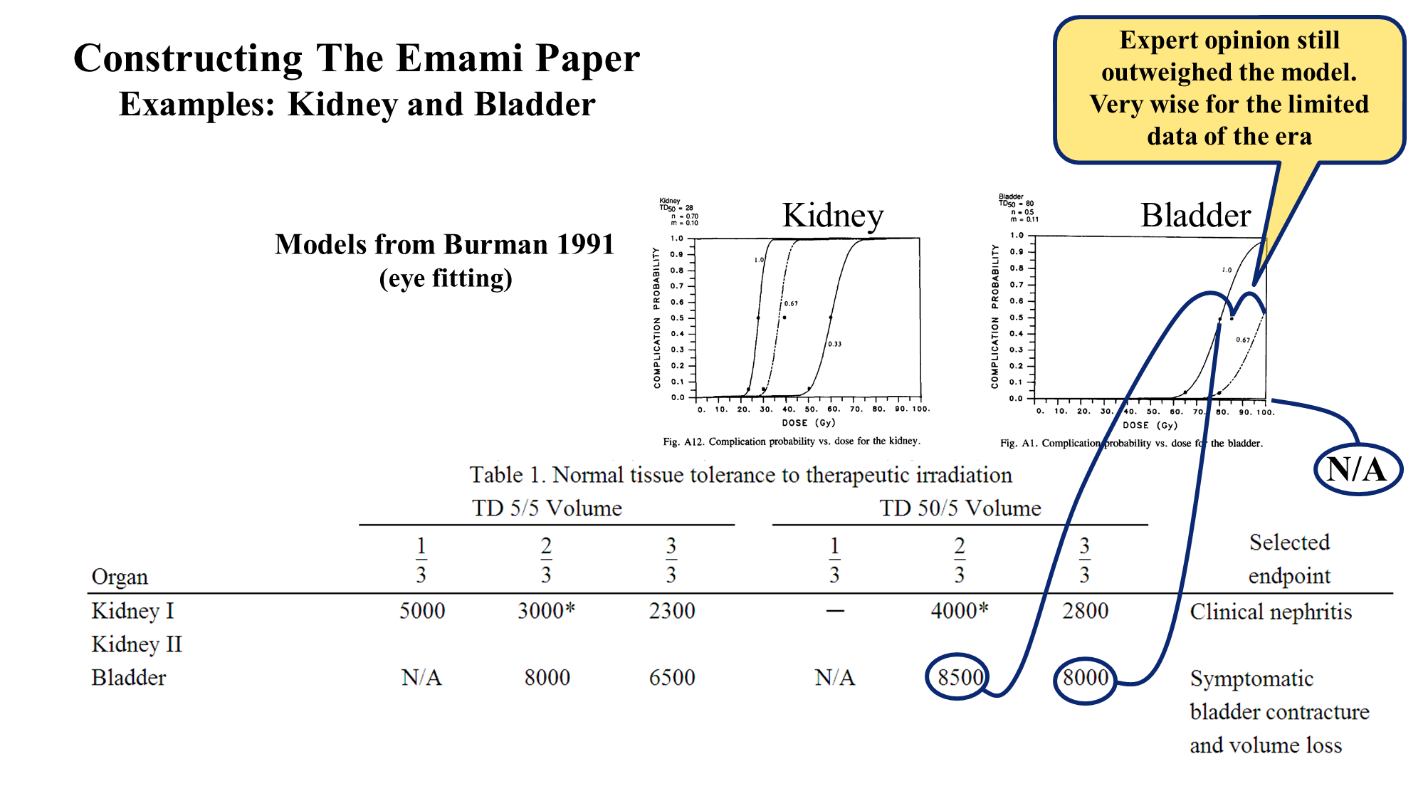


**Fig. A1.** Two examples show the combination of expert opinion and modeling that was used to create the Emami table (34) from the Burman models (35). The dots on the models at the 5% and 50% risk levels predominantly coincide with the numbers in the table, as the 3/3 volume for bladder 50% risk level of 80 Gy is taken directly from the model. Note however, that the model estimated 100 Gy as the 50% risk level for 2/3 of bladder – but expert opinion wisely used 85 Gy in the table instead.


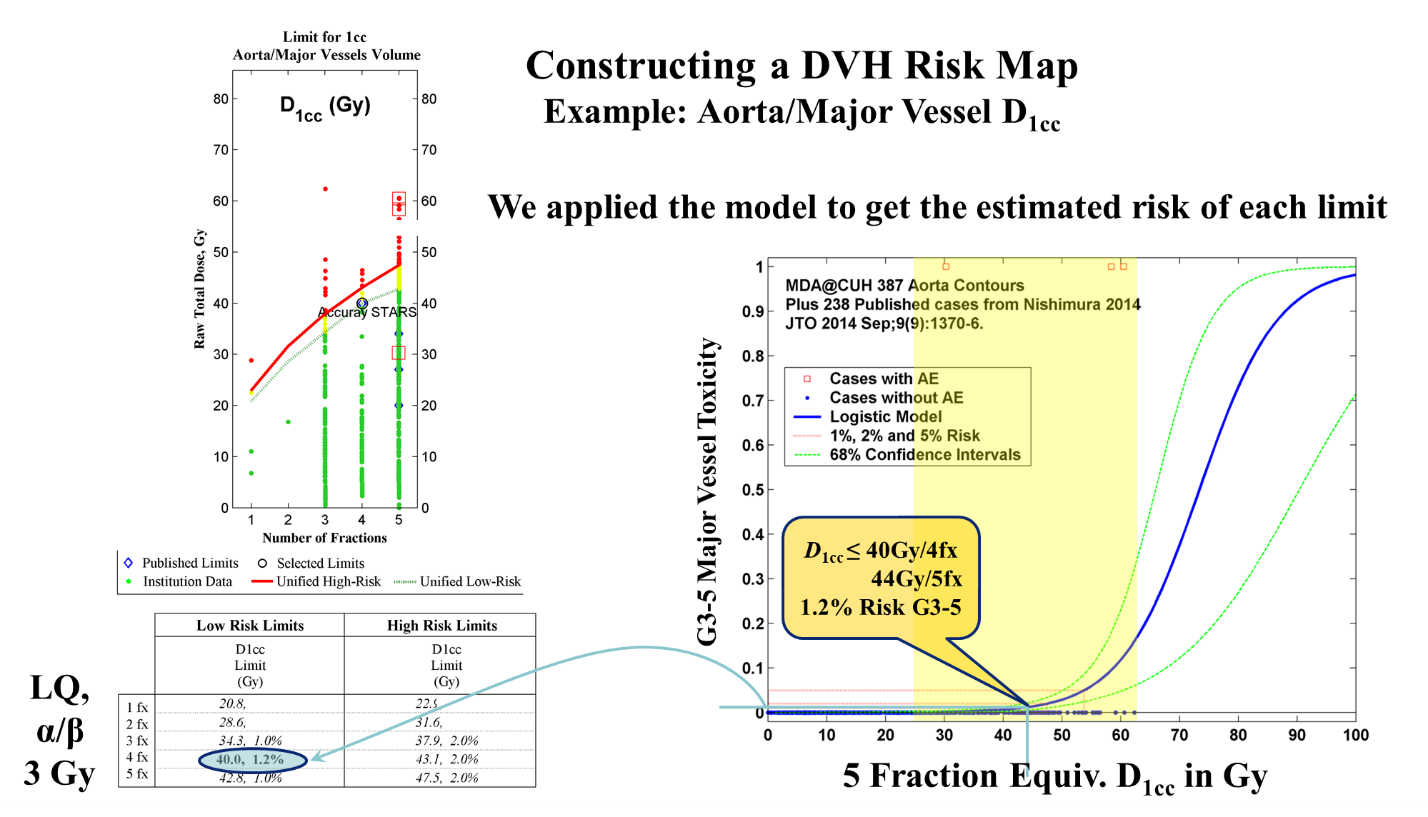


**Fig. A2.** Similar to the Emami table, estimates of risk for each dose tolerance limit in the DVH Risk Map are interpolated from the model. As an example for aorta/major vessel, the D1cc ≤ 40 Gy/4 fraction limit from the Accuray STARS lung protocol (39, 40), has 1.2% risk as estimated from the dose response model. Since the pooled model only contained data in 3-5 fractions (26, 30), risk estimates were not extrapolated to 1-2 fractions. In the DVH Risk Map all estimates of risk are from statistical parameter fitting (21) of dose-response models to clinical outcomes data.


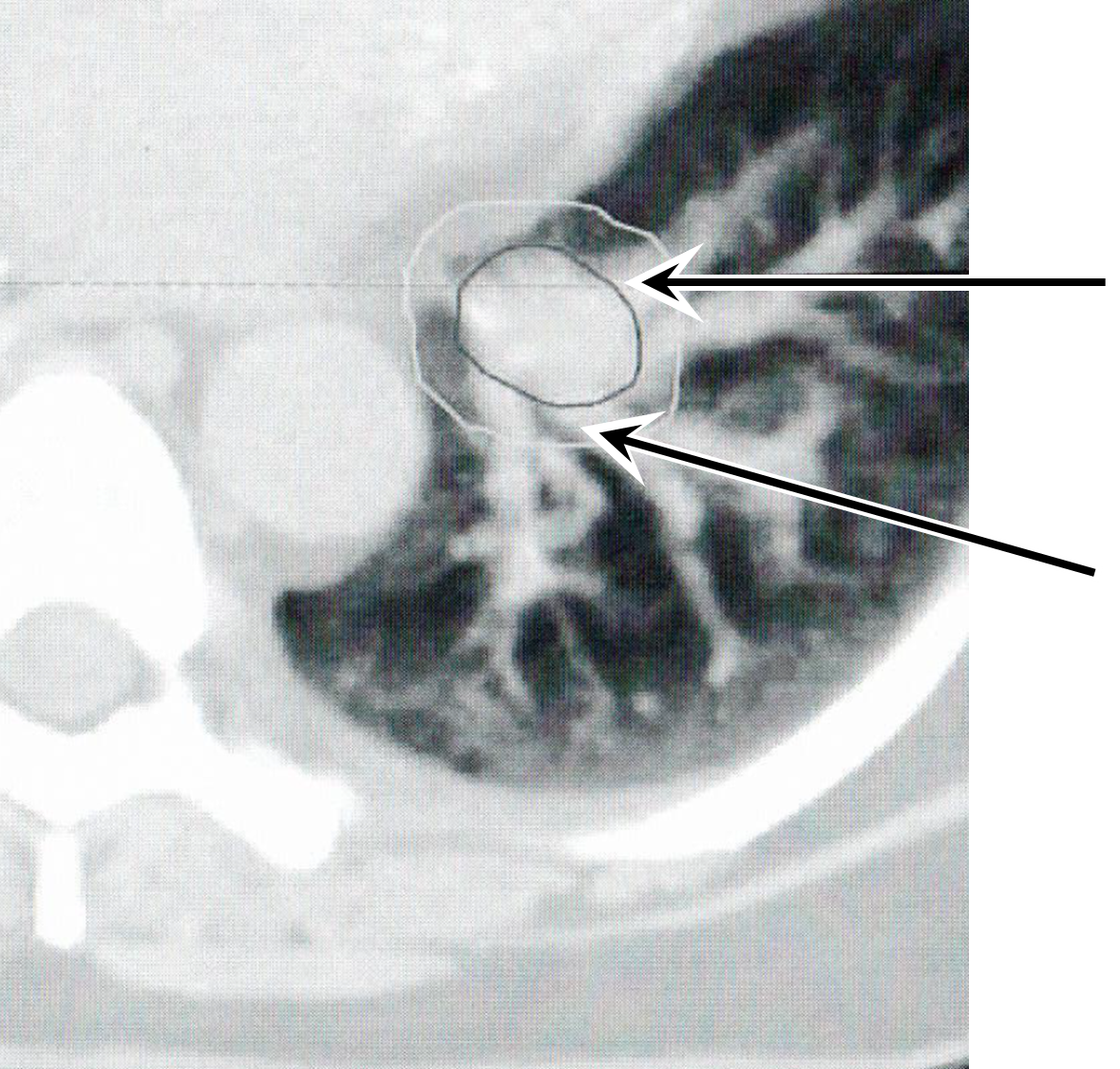


**Fig. A3.** Example of PTV margin overlapping normal tissue, causing areas of concern for the long-term. Increased tracking and targeting accuracy may enable the PTV margins to be smaller, reducing this effect (courtesy of Dr. Bahman Emami, personal communication.) Even a small volume of sensitive structures like bronchus can potentially have long-term complications at such high doses. To quantitatively understand the risk sooner it is important to use models of pooled data from numerous institutions as was done in the Emami Paper (34), QUANTEC (46-47), this paper, and others.


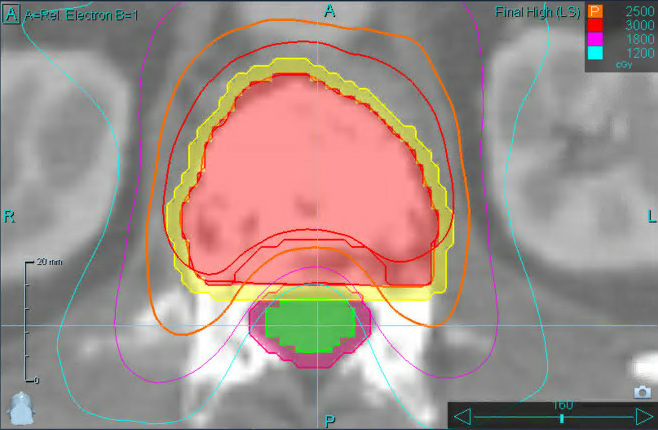

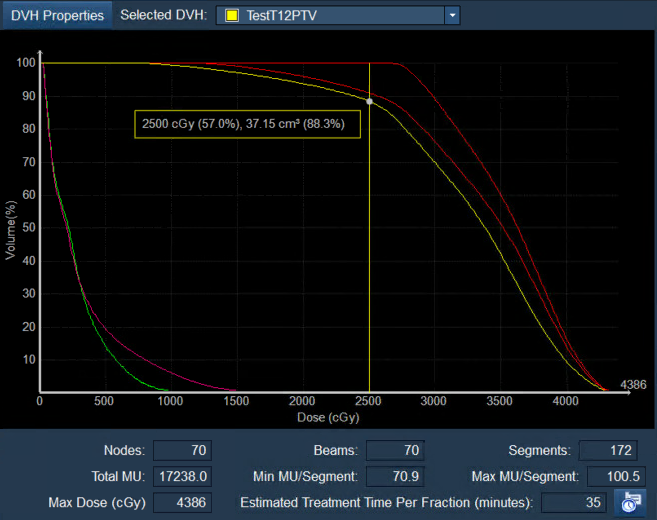


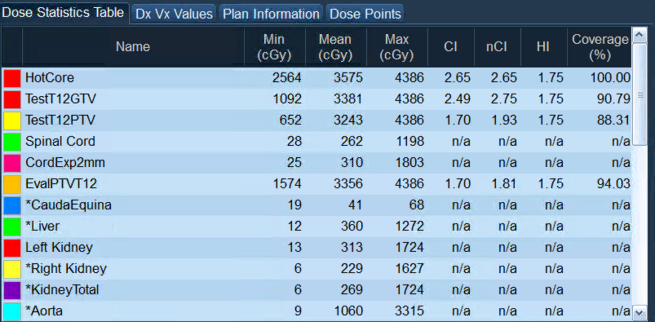

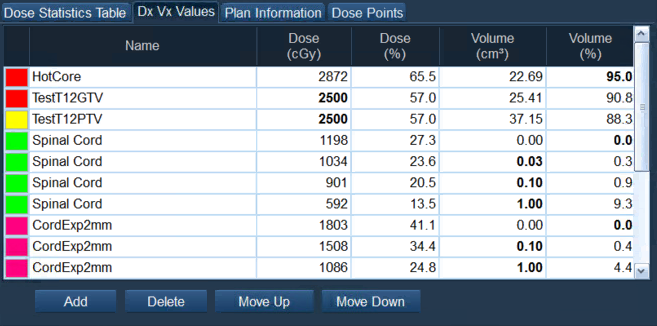


**Fig. A4.** Dose gradient example, spine tumor adjacent to spinal cord. An example treatment plan with 70 non-isocentric beams shows that SBRT with a modern inverse optimization algorithm can achieve such a steep dose gradient that cord+2mm can have 50% higher Dmax than cord. In this case the maximum cord dose is 12 Gy and it is seen that the 1200 cGy isodose line is very conformal to the cord, whereas just 2mm away, the maximum dose to the cord+2mm contour is 50% higher, 18 Gy, and the 1800 cGy isodose line is highly conformal to the cord+2mm contour. The implication is that a 2mm targeting error could result in a delivered cord dose that is 50% higher than the planned dose, underscoring the importance of achieving the best possible targeting accuracy for SBRT.


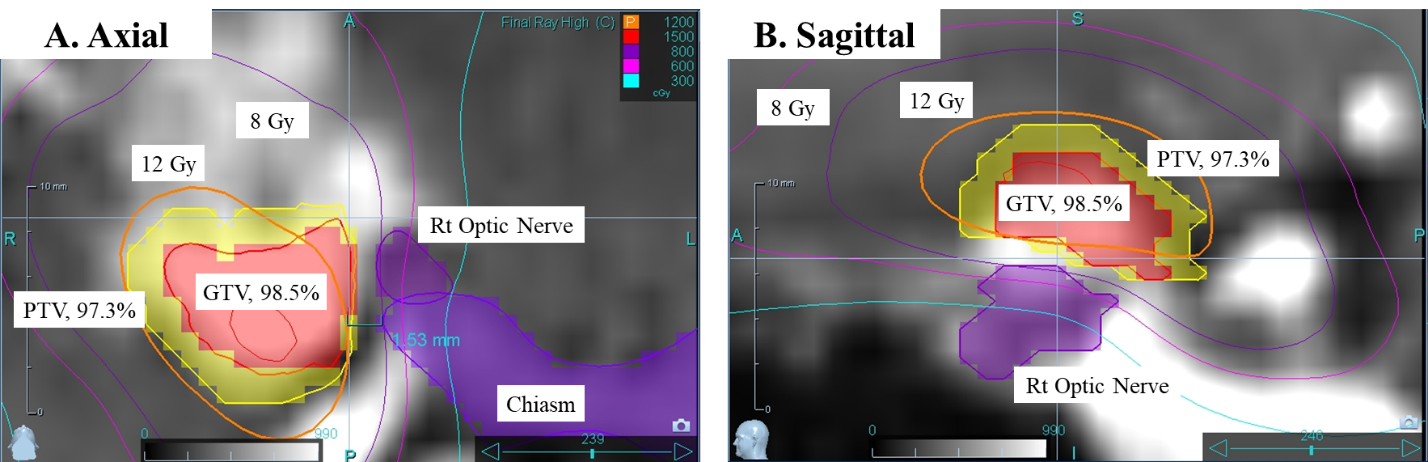


**Fig. A5.** Sphenoid meningioma adjacent to chiasm and optic nerve. Axial (A) and Sagittal (B) planes are shown for a single-fraction plan with 7.5mm and 12.5mm fixed cones, 40 non-isocentric beams, with an estimated 21-minute delivery time. The 12 Gy prescription line is 50% higher than the 8 Gy chiasm limit from Tishler 1993 (48) which is 1.53mm away, comparable to the conceptual example of 50% dose gradient in 1.5mm as in Fig. 4B. This single-fraction plan would have had PTV coverage of 97.3% and GTV coverage 98.5%, but ultimately the physicians decided to use 5 fractions instead so that better tumor coverage would be achievable. Nevertheless, this single-fraction plan is a useful example of the steep dose gradient concept, showing that an extreme such as a 50% dose change is possible within small distances on the order of 1.5mm.
